# Poverty of the Blood

**Published:** 1892-09

**Authors:** 


### POVERTY OF THE BLOOD.

Paleness, thinness of body, weakness and nervousness, are signs of poverty of the blood, or what physicians term anæmia. In some cases palpitation of the heart is often complained of; and when the poverty is the greatest, the lips are pallid and the tongue almost colorless.

In olden times iron was almost wholly relied upon to overcome these symptoms, and at the present day this is the one remedy to which patients suffering from them resort to when they undertake to treat themselves. But physicians, while giving it in many cases, depend far more upon simpler and more effectual measures. These are dietetic and hygienic.

They insist upon free exercise, and that several hours be spent in the open air, either walking or riding, each day. Also at such times that the so-called "breathing exercise" be frequently employed. In the simplest form of this, the subject, while standing with shoulders thrown back, inflates the lungs to the fullest extent, the mouth the meanwhile being closed, and the air entering only through the nose. Of all measures this is one of the most important, for by the means of it the blood is purified and vitalized.

Sponging the body with water that has been made comfortably warm, the operation to be followed by vigorous rubbing with an ordinary towel, is another measure of no little importance, for by it the waste avenues in the skin are kept well open, and, besides, a general tonic effect is secured. Disregard this or other as efficient means for promoting cleanliness, and the blood can never be perfectly pure, for it is sure to take up and carry with it some of the waste matters that should have been expelled through the pores of the skin.

The clothing must be carefully looked to by this class of patients, while in winter "bundling up" is not to be encouraged, yet the clothing should be ample, and that worn next to the skin be of "all wool."

As regards the diet, it should be as highly nutritious as the digestive organs will warrant, and should consist largely of milk, fresh eggs, and beefsteak.

If all these measures are faithfully applied, infinitely much will be done toward restoring the blood and renewing the strength of the system. And if at the same time small doses of iron are taken, the gain from week to week ought to be noticeable. But from this remedy alone very little can be expected.

Considering the fact that very many persons afflicted with the trouble insist upon treating themselves, it is necessary to urge the infinite importance of a physician's advice. It is needed in all cases, but more when the patients are young, and first if they are girls at school. These latter are especially liable to lose ground rapidly, and finally go into consumption.—*Ex*.

